# Gender-based differences of post COVID-19 suicidal ideation among the university students of southern part of Bangladesh: A cross-sectional study

**DOI:** 10.1097/MD.0000000000047837

**Published:** 2026-02-28

**Authors:** Sabiha Shirin Sara, Chuton Deb Nath, Md Asikur Rahman, Riaz Rahman, Ashis Talukder

**Affiliations:** a Statistics Discipline, Science Engineering and Technology School, Khulna University, Khulna, Bangladesh; b Mass Communication and Journalism Discipline, Khulna University, Khulna, Bangladesh; c Department of Applied Epidemiology, National Centre for Epidemiology and Population Health, Australian National University, Canberra, Australia.

**Keywords:** COVID-19, gender differences, mental health, sleep duration, suicidal ideation, university students

## Abstract

Suicidal ideation (SI) represents a manifestation of self-destructive thoughts and behaviors with significant public health implications. This study aimed to determine the prevalence of SI and identify associated risk factors among male and female university students in southern Bangladesh. Data were collected through simple random sampling between April and June 2022 from university students in southern Bangladesh, yielding a final sample of 584 participants (51.5% male, 48.5% female). SI was assessed as the primary outcome variable. Statistical analyses included frequency distributions, chi-square tests, and binary logistic regression to determine prevalence rates and identify associated risk factors. The prevalence of SI was substantially higher among female participants (31.4%) compared to their male counterparts (9.0%). Among female participants, significant risk factors included sleeping <7 hours per night (odds ratio (OR) = 7.670, *P* = .020), having a positive relationship with a partner (OR = 0.336, *P* = .018), experiencing mild (OR = 0.073, *P* < .01) or moderate depression (OR = 0.259, *P* = .014), and reporting no anxiety (OR = 0.031, *P* = .002). For male participants, significant associations were found with having no anxiety (OR = 0.014, *P* = .004) and experiencing moderate anxiety (OR = 0.043, *P* = .009). Female university students demonstrated a significantly higher risk of SI, underscoring the critical importance of gender-sensitive mental health interventions. Findings suggest that female students should be encouraged to maintain regular sleep patterns and cultivate healthy interpersonal relationships. Mental health screening and support services should be prioritized for both genders, with particular attention to depression and anxiety management.

## 1. Introduction

Suicidal ideation (SI) encompasses distressing and complex thought patterns involving contemplation of ending one’s own life.^[1]^ Globally, suicide claims >703,000 lives annually, representing the fourth leading cause of death among individuals aged 15 to 29 years in 2019.^[2]^ The Global Burden of Disease Study identified self-harm as the third leading cause of disability-adjusted life years, among adolescents aged 10 to 24 years in 2019.^[3]^ In 2016, age-standardized suicide mortality rates demonstrated substantial regional variation, with the highest rates observed in Eastern Europe (27.5 per 100,000), followed by the Asia Pacific region (18.7), Central Europe (13.0), North America (12.7), and Australasia (10.6).^[4]^ Notable gender disparities were evident, with Eastern European men exhibiting the highest rate (50.0 per 100,000), while South Asian women demonstrated the highest female rate (12.5 per 100,000).^[4]^

University and college students have been consistently identified as particularly vulnerable populations with respect to SI and behavior.^[5,6]^ A multinational investigation across 12 countries revealed that approximately 29% of university students reported suicidal thoughts, with 7% reporting suicide attempts.^[7]^ In the United States, suicide represents the second leading cause of death among college students, accounting for an estimated 1100 student deaths annually.^[8]^ Within Asia, prevalence estimates demonstrate considerable heterogeneity. A study encompassing students from 6 Association of Southeast Asian Nations countries reported an 11.7% prevalence of SI.^[9]^ In China, prevalence was documented at 7.6% during early university years, increasing to 10% in later academic stages.^[10]^ Among Indian undergraduates, estimates ranged from 3.74% to 13.08%.^[11]^

Multiple mental health conditions – including bipolar disorder, antisocial personality disorder, anxiety disorders, substance use disorders, chronic depression, and dementia, – have been recognized as significant contributors to suicidal behavior.^[12–14]^ Beyond psychiatric disorders, various social determinants exert substantial influence. In the United States, financial stress has been identified as a major risk factor for suicide.^[15,16]^ Evidence suggests that young people in low- and middle-income countries experience elevated rates of SI, influenced by distinct psychosocial challenges and limited access to mental health services.^[17,18]^

In Bangladesh, suicide has emerged as a pressing public health concern, with university students identified as one of the most affected demographic groups, particularly following the coronavirus disease 2019 pandemic. Research indicates that 12.8% of university students in Bangladesh experience suicidal thoughts.^[19,20]^ Alarming trends have been documented, including 5 university student deaths by suicide every ten days^[21]^ and thirteen medical student deaths every twenty-three days.^[22]^ Gender differences are pronounced; overall prevalence among students is estimated at 14.5%, with 12.4% among males and 17.3% among females.^[23]^ Studies indicate higher rates of SI among female students, frequently linked to family-related depression as a primary risk factor.^[24,25]^

Multiple stressors contribute to SI among Bangladeshi university students, including academic pressure, intense competition, personal and emotional difficulties, relationship problems, financial stress, and challenges adapting to the university environment.^[20]^ Despite these concerns, research on SI among Bangladeshi students remains limited, particularly in the southern regions of the country. To address this knowledge gap, the present study aimed to examine and compare the prevalence of SI between male and female university students in southern Bangladesh and to identify key risk factors associated with these behaviors.

## 2. Materials and methods

### 2.1. Sampling and participants

Data were collected using simple random sampling from university students in southern Bangladesh between April and June 2022. Sample size determination employed the Morgan formula:


$$\begin{array}{l} s=\frac{\chi^{2}Np\left(1-p\right)}{d^{2}\left(N-1\right)+\chi^{2}p\left(1-p\right)}=378 \end{array}$$

Where:

- *s* = minimum required sample size,
- *χ*^2^ = Tabulated chi-square value at df = 1 for 0.05 significance level = 3.841,
- N = Total population (25,000),
- *p* = Population proportion (0.5),
- *d* = Degree of accuracy (0.05).

Following the formula calculation, the minimum required sample size was determined to be 378. To achieve this target, a comprehensive list of registered university students was obtained from the registrar office, establishing a population size of 34,000. A sampling frame was created by assigning sequential numbers from 1 to 34,000, and a random number generator was employed to select 600 participants. Among the 600 initially selected participants, 11 students declined participation (response rate: 98.2%), and 5 students were unwilling to complete all survey questions. After accounting for nonresponse and missing data, the final analytical sample comprised 584 participants (51.5% male, 48.5% female).

## 3. Measures

### 3.1. Independent variables

The study examined multiple sociodemographic and health-related variables as potential predictors of SI, including gender, education level, marital status, family income, residence, physical activity, smoking habits, sleep quality, relationship with partner, anxiety, and depression. Marital status was categorized into 3 groups: unmarried, married, and widowed/divorced. Sleep quality was assessed based on average nightly sleep duration and categorized as normal (7–9 hours), less than average (<7 hours), or more than average (>9 hours).^[19]^

### 3.2. Patient health questionnaire (PHQ-9)

Depression was assessed using the PHQ-9, a validated multiple-choice self-report instrument comprising 9 items rated on a 4-point Likert scale ranging from 0 (not at all) to 3 (nearly every day). Total scores range from 0 to 27 and were categorized into 4 severity levels: no depression (<1), mild/minimal depression (1–9), moderate depression (10–14), and severe depression (≥15), combining the originally separate “moderately severe” (15–19) and “severe” (20–27) categories.^[26]^ The PHQ-9 demonstrated acceptable internal consistency in this sample (Cronbach α = 0.786).

### 3.3. Generalized anxiety disorder scale (GAD-7)

The GAD-7, an easily administered self-report questionnaire, served as both a screening tool and severity measure for generalized anxiety disorders. This 7-item instrument employs the 4-point Likert scale ranging from 0 (not at all) to 3 (nearly every day), yielding total scores from 0 to 21. Scores were interpreted as indicating no/minimal anxiety (0–4), mild anxiety (5–9), moderate anxiety (10–14), or severe anxiety (15–21).^[27]^ The GAD-7 demonstrated good internal consistency in this study (Cronbach α = 0.851).

### 3.4. Outcome variable: SI

SI was assessed using the Suicide Behaviors Questionnaire-Revised, the 4-item instrument evaluating different dimensions of suicidality. Item 1 assesses lifetime suicide ideation and/or attempts, Item 2 evaluates frequency of SI over the past twelve months, Item 3 examines threats of suicide attempts, and Item 4 measures self-reported likelihood of future suicidal behavior. The widely validated cutoff score ≥ 7 was employed to identify participants with SI.^[28]^ The Suicide Behaviors Questionnaire-Revised demonstrated acceptable reliability in this sample (Cronbach α = 0.78).

### 3.5. Statistical analysis

Data were initially entered into Microsoft Excel and subsequently analyzed using Statistical Package for the Social Sciences Version 25.0 (Armonk). Analytical procedures included univariate analysis (frequency distributions), bivariate analysis (chi-square tests), and multivariable analysis (binary logistic regression) to identify factors associated with SI stratified by gender.

### 3.6. Ethical approval

All study procedures adhered to ethical standards outlined in the Declaration of Helsinki (1975, as revised in 2008) and were approved by the Research and Innovation Centre at Khulna University (KUECC-2023-05-30), Khulna, Bangladesh. Written informed consent was obtained from all participants, with explicit acknowledgment of their right to withdraw at any time without penalty. The research team conducted ethical evaluations throughout the data collection process in accordance with local laws and institutional regulations.

## 4. Results

### 4.1. Descriptive statistics

Table 1 presents the frequency distributions of all covariates and the dependent variable stratified by gender. The prevalence of SI was substantially higher among female participants (31.4%) compared to male participants (9.0%). Geographic distribution showed that 53.4% of female participants resided in rural areas, whereas 56.5% of male participants lived in urban settings. Physical inactivity was prevalent among both groups, affecting 80.9% of female and 71.1% of male participants.

**Table 1:** Compared frequency distribution table of suicidal ideation among females and male.

Variables	Categories	Female		Male	
		Frequency	Percentages	Frequency	Percentages
Education level	Undergrad (1^st^ yr)	72	25.4	67	22.3
	Undergrad (2^nd^ –4^th^ yr)	182	64.3	210	69.8
	Graduated/postgraduation level	29	10.2	24	8.0
Marital status	Unmarried	246	86.9	292	97.0
	Married	33	11.7	7	2.3
	Divorcee/widowed	4	1.4	2	0.7
Family income	<20,000	121	42.8	120	39.9
	20,000–35,000	97	34.3	89	29.6
	35,000–50,000	39	13.8	59	19.6
	>50,000	26	9.2	33	11.0
Residence	Rural	151	53.4	170	43.5
	Urban	132	46.6	131	56.5
Physical activity	No	229	80.9	214	71.1
	Yes	54	19.1	87	28.9
Smoking habit	No	262	92.6	261	86.7
	Yes	21	7.4	40	13.3
Sleeping h	<7 h	137	48.4	137	45.5
	7–9 h	123	43.5	148	49.2
	>9 h	23	8.1	16	5.3
Relationship with partner	Bad	35	12.4	40	13.3
	Good	188	66.4	176	58.5
	Don’t have	60	21.2	85	28.2
Depression	No depression	3	1.1	4	1.3
	Mild or minimal	155	54.8	192	63.8
	Moderate	79	27.9	73	24.3
	Severe	46	16.3	32	10.6
Anxiety	No or minimal	69	24.4	116	38.5
	Mild	126	44.5	126	41.9
	Moderate	58	20.5	38	12.6
	Severe	30	10.6	21	7
Suicidal ideation	No	194	68.6	274	91.0
	Yes	89	31.4	27	9.0

Regarding sleep patterns, 48.4% of female participants reported sleeping <7 hours nightly, while 49.2% of male participants maintained 7–9 hours of sleep. Depression and anxiety levels differed by gender; severe depression was more prevalent among females (16.3%) compared to males (10.6%), as was severe anxiety (10.6% vs 7.0%, respectively). The majority of participants from both genders were enrolled in their second through fourth years of undergraduate study (69.8% male, 64.3% female), and most reported monthly family incomes below 20,000 Bangladeshi taka.

### 4.2. Bivariate associations

Table 2 presents bivariate associations between SI and selected variables, stratified by gender. Among both male and female participants, marital status, smoking habits, sleep duration, relationship with partner, depression, and anxiety demonstrated significant associations with SI. Family income showed a significant association with SI among female participants (*χ*^2^ = 15.947, *P* = .001) but not among males.

**Table 2:** Compared the bivariate association table among SI and the selected variables.

Variable	Category	Female				Male			
		Suicidal ideation		Chi-square	*P*-value	Suicidal ideation		Chi-square	*P*-value
		No (%)	Yes (%)			No (%)	Yes (%)		
Education level	Undergrad (1^st^ yr)	54 (27.8)	18 (20.2)	2.149	.342	60 (21.9)	7 (25.9)	0.742	.690
	Undergrad (2^nd^ -4^th^ yr)	122 (62.9)	77 (67.4)			193 (70.4)	17 (63.0)		
	Graduated/postgraduation level	18 (9.3)	11 (12.4)			21 (7.7)	3 (11.1)		
Marital status	Unmarried	179 (92.3)	67 (75.3)	15.994	**<.01**	268 (97.8)	24 (88.9)	10.243	**.006**
	Married	14 (7.2)	19 (21.3)			4 (1.5)	3 (11.1)		
	Divorcee/widowed	1 (0.5)	3 (3.4)			2 (0.7)	0 (0.0)		
Family income	<20,000	92 (47.4)	29 (32.6)	15.947	**.001**	107 (39.1)	13 (48.1)	1.954	.582
	20,000–35,000	70 (36.1)	27 (30.3)			81 (29.6)	8 (29.6)		
	35,000–50,000	21 (10.8)	18 (20.2)			54 (19.7)	5 (18.5)		
	>50,000	11 (5.7)	15 (16.9)			32 (11.7)	1 (3.7)		
Residence	Rural	84 (43.3)	48 (53.9)	2.771	.096	120 (43.8)	11 (40.7)	0.093	.760
	Urban	110 (56.7)	41 (46.1)			154 (56.2)	16 (59.3)		
Physical activity	No	36 (18.6)	18 (20.2)	0.110	.740	80 (29.2)	7 (25.9)	0.128	.721
	Yes	158 (81.4)	71 (79.8)			194 (70.8)	20 (74.1)		
Smoking habit	No	6 (3.1)	15 (16.9)	16.818	**<.01**	33 (12.0)	7 (25.9)	4.110	**.043**
	Yes	188 (96.9)	74 (83.1)			241 (88.0)	20 (74.1)		
Sleeping h	<7 h	100 (51.5)	23 (25.8)	16.925	**<.01**	143 (52.2)	5 (18.5)	11.227	**.004**
	7–9 h	79 (40.7)	58 (65.2)			117 (42.7)	20 (74.1)		
	>9 h	15 (7.7)	8 (9.0)			14 (5.1)	2 (7.4)		
Relationship with partner	Bad	6 (3.1)	29 (32.6)	54.673	**<.01**	26 (9.5)	14 (51.9)	38.403	**<.01**
	Good	150 (77.3)	38 (42.7)			168 (61.3)	8 (29.6)		
	Don’t have	38 (19.6)	22 (24.7)			80 (29.2)	5 (18.5)		
Depression	No depression	2 (1.0)	1 (1.1)	77.315	**<.01**	4 (1.5)	0 (0.0)	54.515	**<.01**
	Mild or minimal	138 (71.1)	17 (19.10			185 (67.5)	7 (25.9)		
	Moderate	42 (21.6)	37 (41.6)			67 (24.5)	6 (22.2)		
	Severe	12 (6.2)	34 (38.2)			18 (6.6)	14 (51.9)		
Anxiety	No or minimal	66 (34.0)	3 (3.4)	67.121	**<.01**	114 (41.6)	2 (7.4)	69.630	**<.01**
	Mild	94 (48.5)	32 (36)			122 (44.5)	4 (14.8)		
	Moderate	27 (3.6)	31 (34.8)			27 (9.9)	11 (40.7)		
	Severe	7 (3.6)	23 (25.8)			11 (4.0)	10 (37)		

The bold values have the significance of .01.

SI = suicidal ideation.

Unmarried participants exhibited the highest prevalence of SI in both groups (female: 75.3%, *χ*^2^ = 15.994, *P* < .01; male: 88.9%, *χ*^2^ = 10.243, *P* = .006). Among female participants with family incomes below 20,000 taka, 32.6% reported SI. Smoking was associated with SI in 83.1% of female and 74.1% of male participants.

Notably, 42.7% of female participants with SI maintained good relationships with their partners (*χ*^2^ = 54.673, *P* < .01), whereas among male participants, the highest percentage (51.9%) was observed among those with poor partner relationships (*χ*^2^ = 38.403, *P* < .01). Among female participants with moderate depression, 41.6% reported SI (*χ*^2^ = 77.315, *P* < .01), while among males, the highest percentage (51.9%) was found among those with severe depression. Furthermore, moderate anxiety was associated with SI in 34.8% of females and 40.7% of males.

### 4.3. Multivariable logistic regression analysis

Table 3 presents results from binary logistic regression analyses examining the simultaneous impact of multiple risk factors on SI, stratified by gender. Among female participants, sleep duration, relationship with partner, depression, and anxiety emerged as significant predictors of SI. By contrast, among male participants, only anxiety demonstrated significant associations.

**Table 3:** Compared scenario of SI using binary logistic regression analysis.

Variable	Category	Female				Male			
				95% CI				95% CI	
		OR	*P*-value	Lower	Upper	OR	*P*-value	Lower	Upper
Education level	Undergrad (1^st^ yr)	0.542	.421	0.122	2.411	1.049	.970	0.087	12.661
	Undergrad (2^nd^–4^th^ yr)	0.440	.212	0.121	1.598	0.909	.932	0.103	8.022
	Graduated/postgraduation level	**Ref**							
Marital status	Unmarried	0.455	.635	0.018	11.757	0.551	.999	0.109	3.590
	Married	8.735	.222	0.269	283.168	0.214	.999	0.115	4.445
	Divorcee/widowed	**Ref**							
Family income	<20,000	0.412	.225	0.099	1.725	4.861	.243	0.341	69.265
	20,000–35,000	0.362	.170	0.085	1.544	9.216	.101	0.648	130.99
	35,000–50,000	0.637	.570	0.134	3.027	2.633	.483	0.176	39.369
	>50,000	**Ref**							
Residence	Rural	0.891	.791	0.379	2.093	0.261	.088	0.056	1.220
	Urban	Ref							
Physical activity	No	1.533	.377	0.595	3.954	1.030	.965	0.273	3.887
	Yes	**Ref**							
Smoking habit	No	1.729	.537	0.303	9.849	2.389	.267	0.514	11.113
	Yes	Ref							
Sleeping h	<7 h	7.670	.020	1.376	42.757	0.637	.712	0.058	7.015
	7–9 h	1.821	.500	0.320	10.370	0.147	.161	0.010	2.149
	>9 h	**Ref**							
Relationship with partner	Bad	2.347	.199	0.638	8.631	4.879	.062	0.926	25.710
	Good	0.336	.018	0.136	0.832	0.717	.686	0.143	3.606
	Don’t have	**Ref**							
Depression	No depression	6.625	.256	0.254	172.666	0.618	.688	0.059	6.479
	Mild or minimal	0.073	<.01	0.022	0.243	0.260	.173	0.037	1.807
	Moderate	0.259	.014	0.088	0.760	0.221	.165	0.124	2.227
	Severe	**Ref**							
Anxiety	No or minimal	0.031	.002	0.003	0.283	0.014	.004	0.001	0.262
	Mild	0.433	.176	0.129	1.457	0.043	.009	0.004	0.463
	Moderate	0.819	.750	0.239	2.802	0.400	.342	0.060	2.651
	Severe	**Ref**							

CI = confidence interval, SI = suicidal ideation.

Female participants who maintained good relationships with their partners were 66.4% less likely to experience SI compared to those without partners (odds ratio [OR] = 0.336, 95% confidence interval [CI] = 0.136–0.832, *P* = .018). Female participants with mild depression were 92.7% less likely (OR = 0.073, 95% CI = 0.022–0.243, *P* < .01), and those with moderate depression were 74.1% less likely (OR = 0.259, 95% CI = 0.088–0.760, *P* = .014) to report SI compared to participants with severe depression.

Regarding anxiety, female participants reporting no/minimal anxiety were 96.9% less likely to experience SI (OR = 0.031, 95% CI = 0.003–0.283, *P* = .002) compared to those with severe anxiety. Similarly, male participants with no/minimal anxiety were 98.6% less likely (OR = 0.014, 95% CI = 0.001–0.262, *P* = .004), and those with moderate anxiety were 95.7% less likely (OR = 0.043, 95% CI = 0.004–0.463, *P* = .009) to report SI compared to those with severe anxiety.

Female participants sleeping <7 hours nightly demonstrated significantly elevated odds of SI (OR = 7.670, 95% CI = 1.376–42.757, *P* = .020) compared to those sleeping >9 hours.

## 5. Discussion

SI exerts profound and widespread effects on individuals, families, communities, and nations. Research indicates that suicide attempt rates peak among individuals in their mid-to-late twenties.^[29]^ The primary objective of this investigation was to examine the prevalence of SI among university students in southern Bangladesh and to assess whether gender influenced varying levels of SI.

### 5.1. Principal findings

Our research revealed a substantial gender disparity in SI among university students in the southern region of Bangladesh. Approximately 9.0% of male students and 31.4% of female students reported experiencing SI. This finding aligns with previous studies indicating elevated susceptibility to suicide among females.^[19,20,30]^ Gender-related vulnerabilities to psychopathology or psychosocial stressors may contribute to the increased prevalence of SI among female students.^[31]^

### 5.2. Sleep duration and SI

A particularly noteworthy finding was that short sleep duration (<7 hours nightly) demonstrated a significant association with SI exclusively among female participants, with no significant correlation observed among males. This relationship between sleep disruption and increased risk of SI may be attributed to sleep deprivation potential to exacerbate impulsive and aggressive behaviors, as well as emotional instability.^[32]^

### 5.3. Interpersonal relationships

Maintaining positive relationships with romantic partners was found to reduce the risk of SI among female participants compared to those without partners. This protective effect may be linked to stress reduction, enhanced self-confidence, alleviation of loneliness, and improved overall mental well-being.^[33]^

### 5.4. Depression and gender

Depression demonstrated a significant association with SI exclusively among female participants. Specifically, females experiencing mild or moderate depression were 92.7% and 74.1% less likely, respectively, to report SI compared to those with severe depression. This gender-specific pattern may be attributed to females’ tendency to manifest internalizing symptoms, heightened sensitivity to interpersonal relationships, and susceptibility to specific depression-related conditions.^[34,35]^

### 5.5. Anxiety across genders

Anxiety, as a mental health condition, exhibited significant associations with SI in both male and female participants. Compared to those with severe anxiety, individuals with no/minimal or mild anxiety demonstrated a substantially lower likelihood of reporting SI. Factors including personal, social, and academic transitions, as well as cyberbullying, may contribute to elevated anxiety levels, potentially increasing the risk of SI.^[36]^

### 5.6. Socioeconomic factors

Family income demonstrated significance in relation to SI exclusively among female participants in bivariate analysis. Consistent with prior research, higher annual household income appeared to reduce the risk of SI.^[37]^ However, our findings diverged from a Korean study that identified significant associations between SI and medium-high income groups.^[38]^

### 5.7. Smoking behavior

Smoking status emerged as a factor associated with elevated risk of SI among both males and females. Potential causal mechanisms linking smoking to SI include depression, neurochemical alterations, and the development of smoking-related health conditions such as lung cancer.^[33]^

### 5.8. Clinical and integrative care implications

The study’s findings carry significant implications for clinical practice and students' mental health services. The elevated prevalence of SI among female university students, coupled with its associations with sleep duration, relationship quality, depression, and anxiety, underscores the necessity for gender-sensitive and integrative care approaches. Routine mental health screening in university health settings should incorporate assessments of sleep patterns, emotional well-being, anxiety symptoms, and psychosocial stressors, with particular attention to female students.

An integrative paradigm incorporating collaboration among medical specialists, mental health practitioners, counselors, and student support services may facilitate early identification of at-risk individuals and enable timely intervention. Such comprehensive, multidisciplinary approaches may contribute to improved mental health outcomes and reduced SI among university populations.

### 5.9. Strengths and limitations

This study’s strengths include its random sampling methodology, substantial sample size, use of validated instruments, and focus on an understudied population in southern Bangladesh. However, several limitations warrant consideration. The cross-sectional design precludes causal inference. Self-reported data may be subject to recall and social desirability biases. The study was conducted during the coronavirus disease 2019 pandemic, which may have influenced mental health outcomes. Additionally, the findings may not be generalizable to university students in other regions of Bangladesh or other countries.

## 6. Conclusion

This study revealed substantial gender disparities in SI among university students in southern Bangladesh, with female participants demonstrating a prevalence rate (31.4%) >3 times higher than that of male participants (9.0%). For female students, sleep duration, relationship quality with partners, depression severity, and anxiety emerged as significant risk factors for SI. Among male participants, only anxiety levels demonstrated significant associations with SI.

These findings underscore the critical need for enhanced attention to female students’ mental health, as they face a substantially higher risk of SI compared to their male counterparts. Mental health awareness and intervention programs should address both depression and anxiety, which demonstrated significant associations with SI across both genders. Female students should be encouraged to maintain regular sleep patterns and cultivate supportive interpersonal relationships as protective factors against SI.

This investigation contributes to creating an open environment for discussing gender-based differences in SI and empowering individuals to seek help and find hope, ultimately working toward reducing gender-based disparities in SI among university students.

## Acknowledgments

We acknowledge all participants who generously contributed their time to this study.

## Author contributions

**Conceptualization:** Sabiha Shirin Sara, Md Asikur Rahman, Riaz Rahman, Ashis Talukder.

**Data curation:** Sabiha Shirin Sara, Md Asikur Rahman, Riaz Rahman, Ashis Talukder.

**Formal analysis:** Sabiha Shirin Sara, Md Asikur Rahman, Riaz Rahman, Ashis Talukder.

**Funding acquisition:** Sabiha Shirin Sara.

**Methodology:** Sabiha Shirin Sara, Riaz Rahman, Ashis Talukder.

**Validation:** Sabiha Shirin Sara, Chuton Deb Nath, Md Asikur Rahman, Riaz Rahman, Ashis Talukder.

**Visualization:** Sabiha Shirin Sara, Chuton Deb Nath, Md Asikur Rahman, Riaz Rahman, Ashis Talukder.

**Resources:** Chuton Deb Nath.

**Software:** Riaz Rahman, Ashis Talukder.

**Supervision:** Ashis Talukder.

**Writing – original draft:** Sabiha Shirin Sara, Chuton Deb Nath, Md Asikur Rahman, Riaz Rahman, Ashis Talukder.

**Writing – review & editing:** Sabiha Shirin Sara, Chuton Deb Nath, Md Asikur Rahman, Riaz Rahman, Ashis Talukder.
